# Culture and Attention: Future Directions to Expand Research Beyond the Geographical Regions of WEIRD Cultures

**DOI:** 10.3389/fpsyg.2020.01394

**Published:** 2020-07-24

**Authors:** Takahiko Masuda, Batgerel Batdorj, Sawa Senzaki

**Affiliations:** ^1^Department of Psychology, University of Alberta, Edmonton, AB, Canada; ^2^Shine Mongol Harumafuji School, Ulan Bator, Mongolia; ^3^Department of Psychology, University of Wisconsin – Green Bay, Green Bay, WI, United States

**Keywords:** culture, Mongolia, drawing, holistic vs. analytic attention, WEIRD

## Abstract

Henrich et al. (2010) highlighted the necessity of broadening the range of regions for cross-cultural investigation in their seminal paper “The weirdest people in the world.” They criticize the current psychological framework for relying dominantly on American undergraduate students for their participant database, and state that there is a risk associated with investigating human nature by focusing solely on a unique population. This line of research has, over the past 30 years, successfully demonstrated the diversity of human cognition. However, it is true that there are still only a limited number of studies that have extended their geographical regions of research outside of G7 (Canada, France, Germany, Italy, Japan, United Kingdom, and United States) and G20 countries (Argentina, Australia, Brazil, China, India, Indonesia, Mexico, Russia, Saudi Arabia, South Africa, South Korea, Turkey, EU countries, and the above G7 countries). In order to fully examine the issue of culture and cognition, we maintain that the field of psychology must extend its research globally. In this paper, we will briefly discuss the history of cross-cultural research in the 1960s which can be seen as the beginning of addressing the above concerns, and review some contemporary empirical studies which took over their 1960s predecessors’ mission. Here we address three strengths of extending the geographical scope to advance cultural psychology. In the second half of the paper, we will introduce our preliminary study conducted in Mongolia as a sample case study to demonstrate a way of administering cultural psychological research outside of the existing research field. We will then discuss implications of this line of research, and provide tips on how to open a new research site.

## Introduction

A group of researchers in the late 1980s and early 1990s began advocating for the development of a new research field in social sciences in order to understand the human mind in cultural contexts (Bruner, 1990; Shweder, 1991; Miller, 1999). This interdisciplinary field of research, which includes linguistics, psychology, anthropology, and neuroscience, is now known as “cultural psychology.” Researchers in this field challenged the universality of human psychological processes and advocated the importance of understanding human psychology in cultural contexts. Recently, they have developed a variety of theoretical frameworks, devised research methodology and tasks for experimentation, and demonstrated substantial cultural variations in even basic psychological processes—notably human cognition (e.g., Masuda et al., 2019, for review). However, research programs have not fully expanded to new geographical regions. In this paper, we address the need to conduct research outside of G7 (Canada, France, Germany, Italy, Japan, United Kingdom, and United States) and G20 countries (Argentina, Australia, Brazil, China, India, Indonesia, Mexico, Russia, Saudi Arabia, South Africa, South Korea, Turkey, EU countries, and the above G7 countries), and discuss an important direction to further advance research on culture and human cognition. As one example that demonstrates this new way of conducting research, we will also share results from a case study held in Mongolia.

### Patterns of Cognition and Social Orientation

One of the goals of cultural psychologists is to present empirical evidence that the majority of so-called “universal psychological phenomena” reported in North American professional journals are actually the products of mutual constitutions between culture and the mind. For over 30 years, cultural psychologists have examined cultural variations in basic psychological processes between people in East Asian and North American societies in order to address this goal.

A theoretical framework developed by Nisbett et al. (2001), Nisbett (2003), Nisbett and Masuda (2003), Nisbett and Miyamoto (2005), and Masuda et al. (2019), for example, contrasted two patterns of cognition: *analytic* and *holistic. Analytic cognition*, dominant in Western cultures such as Western Europe and North America, is characterized by discourses that emphasize an object-oriented focus in visual attention (selectively focusing more on objects than on context). In contrast, *holistic cognition*, dominant in East Asian cultures such as China, Korea, and Japan, is characterized by discourses that emphasize a context-oriented focus of attention (attending to objects in relation to their context).

Based on this theoretical framework, a substantial number of empirical studies have demonstrated that East Asians are more likely than North Americans to be sensitive to contextual information in many aspects of social cognition (see Masuda, 2017, for review). For example, Masuda and Nisbett (2001) demonstrated that, when asked to describe animated vignettes of underwater scenes, Japanese tended to holistically capture the entire scene, whereas Americans tended to analytically detach the main object in the scene, and selectively describe the features of that object. Implementing the theory of holistic vs. analytic cognition, researchers have documented cultural variations in causal attribution, inference, categorization, spatial and temporal perception, emotion perception, and even artistic expression and design of Internet web pages (Masuda, 2017, for review).

Varnum et al. (2010) further discussed that people’s social orientation—*interdependence vs. independence*—could play an important role for people to develop culturally specific cognitions—*holistic vs. analytic*, respectively. One explanation for the connection between *independence* and *analytic cognition* is that those who live in societies where independent social orientation is dominant find that the world consists of events, people, and objects which are, in general, independent from each other. Such a view facilitates people to selectively pay attention to what they think is the most important event, object, or person in the scene, while intentionally ignoring the context. In contrast, an explanation for the connection between *interdependence* and *holistic cognition* is that those who live in a society where interdependent social orientation is dominant need to become sensitive to other people’s feelings in order to maintain the society. This requires having communal and cooperative relationships. Such a sensitivity to relationships facilitates people to pay attention not only to the target issue but also to its surrounding context. When such social practices are generalized, people tend to use holistic attention, capturing all of the scenes and relationships in a single event.

### The Criticism Against General Psychology: WEIRD People in the World

Recently, a problem was addressed by Henrich et al.’s (2010) seminal paper “The Weirdest People in the World,” in which the authors criticized psychological data reported in major North American journals for relying too much on North American undergraduate students to have their findings naively accepted as universal. Henrich et al. called this population “WEIRD,” which stands for “Western, Educated, Industrialized, Rich, and Democratic.” More specifically, WEIRD people in many cases represent American undergraduate students who have taken psychology courses, and whose contributions make up a major part of the psychological database. Although the database is valuable for advancing psychological science, Henrich et al., 2010 argue that it is not representative of the human population, and therefore cannot be generalized. In fact, the ratio of the WEIRD population to the entire human population is very small, suggesting that WEIRD people are rather unusual outliers.

Why are researchers reluctant to access people outside of the WEIRD category? Possible reasons attributing to the biased data collections include the following: (1) the North American student subject pools at universities allow researchers to easily conduct studies as they are familiar with participating in experiments as their human subjects and there is little cost to using them; (2) the majority of psychologists have trained in North American academic institutions; the Western tradition of scientific investigation is quite analytic (i.e., ignores contexts by assuming they play less important roles in shaping one’s mind); and (3) researchers are not fully aware of the fact that they are culturally biased, and assume that the North American mind is standard, universal, and invariant across all cultures.

### History of Cross-Cultural Investigation in Human Cognition and Perception

Outside psychology, doubts of universality in the mind and the idea that there are substantial variations in psychological processes have been discussed in a broader context in the Humanities and Social Sciences. The history of cross-cultural studies regarding human nature is rather long. For example, the seeds of cultural psychology can be traced back to Wilhelm Wundt’s (1916) “völkerpsychologie” (folk psychology), where he addressed descriptive investigations of cultural phenomena. A variety of fields have also contributed to the study of cultural variations in human nature, including anthropology (e.g., Kluckhohn, 1949; Geertz, 1973) and linguistics (e.g., Whorf, 1956). Furthermore, other domains of psychology including developmental psychology (e.g., Vygotsky, 1978; Luria, 1979), and social psychology (e.g., Sherif, 1936) have also contributed to the study of cultural variations. In the field of cognitive sciences, “New Look Psychology,” advocated by Bruner and Goodman (1947) and Bruner (1957), researchers maintained that even our perception—so called basic psychological processes—is highly influenced by cultural contexts.

While theoretical discussions have been addressed in a variety of fields, empirical studies on cross-cultural variations in basic psychological processes have focused on small-scale societies since the 1960s (for an extensive review see Cole and Scribner, 1974; McCauley and Henrich, 2006). One of the earliest empirical studies where researchers extended their data collection outside the geography of North American laboratories is a series of anthropological studies involving the Müller-Lyer illusion (Rivers, 1901, 1905; Segall et al., 1966). This optical illusion refers to people’s perception of the length of lines where same length lines with inward arrows are judged to be longer than the lines with outward arrows. Segall et al. (1966) demonstrated that the effect of the Müller-Lyer illusion, which had been reported in Western data, was observed in Melanesian and Indian tribal members, but not in a hunter-gatherer group in the Kalahari Desert.

These early studies have maintained that ecological factors—people’s actual life experiences in a particular geographical and ecological environment—are keys to understanding people’s perception and cognition. For example, the “carpenter hypothesis,” maintains that one’s experience of viewing things three dimensionally affects the magnitude of the illusion, especially if the environment contains rectangular architectures. In such an environment, lines with inward arrows often appear as front edges of rectangular shaped buildings, which are closer to the viewer, whereas lines with outward arrows often appear as rear edges, which are farther away from the viewer. Because of this experience, people tend to overestimate the length of inward arrows compared to outward arrows. In other words, the magnitude of the optical illusion is reduced for those who have not experienced such an environment, suggesting that the optical illusion is not innate but shaped by one’s environmental experience (Hudson, 1960, 1962a,b, 1967; Mundy-Castle, 1966; Deregowski, 1968a,b).

In other early research on perception, researchers contrasted perceptual styles in field-dependent vs. field-independent perception (e.g., Witkin et al., 1954; Witkin, 1967; Witkin and Berry, 1975; Witkin and Goodenough, 1977). Using perceptual tasks (e.g., embedded figure task), these researchers investigated individual differences in context sensitivity. For example, field-independent individuals were better at detecting target shapes which were superimposed on multi-layered shapes than field-dependent individuals. These perceptual tasks were later used to identify cultural variations in attention, finding that people in cities, independent cultures, and hunting communities tended to show context independent patterns of perception (Dowson, 1967; Berry, 1971, 1976).

In sum, cross-cultural investigations into human cognition were conducted from the 1960s through to the 1970s, but fell out of favor partially because mainstream North American psychologists began searching for evidence of universality rather than specificity (e.g., Fodor and Pylyshyn, 1981; Fodor, 1983; Pylyshyn, 1999). The contemporary cultural psychology research began in the late 1990s by focusing on empirical investigations and applying some of the classic methods from earlier cross-cultural studies to different populations (e.g., Ji et al., 2000). There is an inherent affinity between contemporary cultural psychology and cross-cultural studies from the 1960s. In line with the WEIRD critics, we maintain that researchers should acknowledge the classic cross-cultural studies in small-scale societies and should reconsider the importance of conducting research outside of G7 and G20 countries.

### Beyond the East vs. West Dichotomy

The idea of holistic cognition/interdependent social orientation vs. analytic cognition/independent social orientation has been applied to many cross-cultural studies of East Asians and North Americans. Why has the East vs. West dichotomy been so often utilized by contemporary cultural psychologists? After Markus and Kitayama’s seminal review paper (1991) was published, the issue of culture caught mainstream psychologists’ attention, which resulted in advancing contemporary cultural psychology for two main reasons: First, going beyond cross-cultural studies of the 1960’s, contemporary psychologists successfully demonstrated that cultural variations in cognition linger even when they target post-industrial societies in East Asia. For example, Japan is one of the G7 countries, of which demographic factors such as the level of education, level of modernization, GDP level, as well as methodological factors such as accessibility to undergraduate participants and school curriculums in colleges are quite comparable to that of North American and European counterparts, yet researchers persuasively demonstrated substantial diversity in cognition between these countries (e.g., Kitayama et al., 1997). Second, from a social sciences perspective, the rapid economic growth in the East Asian economy would also influence mainstream psychologists’ perception about culture. The East/West dichotomy dominated the business discourse of the 1980’s due to economic competition between America and Japan, and it still persists. Retrospectively, it has been one of the factors which indirectly increased the number of audiences who were interested in successful findings in cultural psychology.

However, like Henrich et al. (2010)’s argument regarding the limits of relying on data from WEIRD societies, the East/West dichotomy also limits our scope and forces us to question our ability to claim universality in cognitive processes. There are at least three beneficial points for researchers to further broaden this field of research to go beyond the East/West dichotomy.

First, while a plethora of evidence has depicted differences between the cultures of the two continents of East Asia and North America, researchers need to be prudent not to overgeneralize this dichotomy onto cultures in other continents: there is not much known about Europe, South America, the Middle East, Africa, and Central Asia. It is only recently that researchers have acknowledged the gaps in the data of the global population. For example, Kitayama et al. (2009) demonstrated that British and German participant’s level of holistic attention fell between Americans and Japanese, showing a moderate level of context sensitivity. Meanwhile, all the Western groups showed stronger dispositional biases in person perception compared to Japanese, and in the implicit measurement of interdependence, Japanese scores were higher than the Western populations. Similarly, San Martin et al. (2018) studied Arab populations, and identified that Saudis and Lebanese were as interdependent in their self-perception, holistic in their attention, and context-oriented in their behavioral inference patterns as Japanese, yet they were more assertive in their emotional experiences than Japanese. Uskul et al. (2008) also tested three small communities in the Black Sea region of Turkey. Participants were ethnically similar and spoke the same language but differed in their primary economic activities and subsistence systems. They found that herders, who often work alone, tend to hold a more independent social orientation and analytic thinking style, whereas farmers and fishers, whose cooperative working styles are both valued and required, tend to hold a more interdependent social orientation and holistic thinking style. However, research has scarcely been conducted in other regions such as Central Asia, South America, and Africa, even though there are substantial amounts of ethnography and data provided by anthropologists, sociologists, and regional researchers.

Second, the East-West dichotomy often results in unclear explanations regarding the key variables that directly influence one’s pattern of cognition. For example, Southern Chinese tend to hold holistic patterns of attention, and it is often argued that their rice-farming tradition, which requires intensive collaboration such as creating irrigation systems, is the antecedent of their interdependent social orientation (Talhelm et al., 2014). However, there is still a need for researchers to articulate the causality between culture and cognition, and much research is needed to test the replicability of the findings. Can interdependent social norms be independent from the culture’s dominant economic system? What if the dominant economic system of the target culture is nomadic pastoral (a potential ecological facilitator of analytic cognition), yet they are socially interdependent (a potential factor in making people holistic)? In order to answer these questions, researchers must investigate explanatory variables and scrutinize the magnitude of the effect of each variable.

Third, while cultural psychologists have advocated for many theoretical frameworks predicting cultural variations in human cognition, it is important to further search for variables and dimensions by which researchers can capture a variety of cultural phenomena all over the world. For example, San Martin et al. (2018) identified that interdependence and assertiveness are key variables in explaining Arabs’ mentalities. Where does the assertiveness come from? Are there other cultures which show similar mentalities? What are the effects of religious beliefs (e.g., shamanism, monotheism, polytheism, animism, etc.), political systems (e.g., communistic, socialistic, democratic, etc.), population density (e.g., dense, intermediate, scarce, etc.), or relational mobility—a socioecological variable (Yuki and Schug, 2012) that represents how much freedom and opportunity a society affords individuals to choose and dispose of interpersonal relationships based on personal preference (e.g., high-voluntary vs. low-fixed)? Currently, cultural psychologists can provide few answers to these questions.

In summary, this section has discussed a brief history of the theoretical development of cultural research, classic empirical studies, and the recent advances of research on culture and cognition outside of the WEIRD population and the East-West dichotomy. We also discussed three major reasons for researchers to extend their region of research outside of G7 and G20 countries. In the next section, we will introduce a case study as an example of initiating a cross-cultural study in a new culture.

## Mongolian School-Aged Children and Teenagers’ Landscape Drawing Styles: a Case Study

In this study, we selected Mongolia as our target culture and created networks with Shine Mongol Elementary and Secondary schools, part of the New Mongol Academy, Ulaanbaatar, Mongolia, where we implemented a study using a simple picture drawing task (Masuda et al., 2008). The main objective of this study was to examine Mongolian children and teenager’s patterns of perception. In addition, we aimed to compare the data with an existing cross-cultural dataset (Nand et al., 2014) to examine the similarities and differences in drawing styles across three countries: Mongolia, Canada, and Japan. By doing so, we attempted to demonstrate a way of extending research to a non-WEIRD culture (e.g., Henrich et al., 2010) and strove to go beyond the East-West dichotomy (e.g. Markus and Kitayama, 1991; Nisbett, 2003).

### Mongolian Culture

Ancient historical documents reported that people of many ethnic backgrounds have lived in the geographical area of Mongolia^1^. The earliest report of Mongolians appeared in Chinese documents published in the 11th century, but their activities can be traced back to the 9th century. In the 13th century, Genghis Khan (Chinggis Khaan) founded the Mongol Empire, and his influence spread across Eurasia. From the 17th century, the area was ruled by the Qing dynasty in China, influencing the country until the end of the 19th century. In the early 20th century, the Mongolian People’s Republic was established under the authority of the Soviet Union. The Mongolian people further transformed in the late 1980s and early 1990s, and in 1992, the government issued a new constitution implementing a multi-party system and endorsing a market economy.

Because of their herding and nomadic traditions, family oriented lifestyle, religious beliefs (e.g., Buddhism, Shamanism, and Animism), and the ecology of the area, researchers can speculate a variety of socio-economic-geographical variables which may influence Mongolians’ minds. While acknowledging intra-cultural variations, we assumed there would be a dominant pattern of psychological processes in this culture. Although there are many anthropological and sociological studies (e.g., Fijn, 2011; Sakamoto et al., 2015), psychological studies with empirical methodology have rarely been conducted thus far.

Historically, Mongolian culture has developed independently from WEIRD populations and from East-West contrasts, while they have experienced political influences from Russia and China. Therefore, it is important for cultural psychologists to examine Mongolians and their civilization in order to discover potentially unique characteristics in their mindset. We present two competing hypotheses: (1) since Mongolians value family ties, they hold more interdependent and less independent social orientation (Markus and Kitayama, 1991; Varnum et al., 2010), therefore their patterns of attention are more similar to East Asians than North Americans, or (2) because of their tradition of pastoral and nomadic lifestyle, one that has been said to be relatively independent from any other subsistence system, Mongolians are less holistic and more analytic (Nisbett et al., 2001; Uskul et al., 2008; Masuda et al., 2019), suggesting that their patterns of attention are more similar to North Americans than East Asians. In addition to the need to test the above competing hypotheses, we sought to discover unique characteristics of the Mongolian mind.

The current study chose to implement a landscape drawing task used in previous studies (Masuda et al., 2008; Nand et al., 2014; Senzaki et al., 2014). Those results indicated that East Asians have developed an artistic tradition of expression which emphasizes context, whereas Westerners, especially North Americans, have developed another artistic tradition of expression which emphasizes main objects rather than context. Furthermore, contemporary members of each culture (as opposed to classic painters), such as school-aged children, teenagers, and young adults demonstrate culturally dominant patterns of artistic expressions when asked to draw a landscape image. Masuda et al. maintain that the observed patterns of drawing reflect the dominant pattern of attention: East Asians view things holistically and in a context-oriented manner associated with their sense of interdependent social orientation, whereas North Americans view things analytically and in an object-oriented manner associated with their sense of independent social orientation (Nisbett et al., 2001; Nisbett, 2003; Nisbett and Masuda, 2003; Masuda, 2017; Masuda et al., 2019).

We focused selectively on school-aged children because we assumed that cultural variation in cognition is attributable to one’s internalization and socialization of a culturally shared meaning system (Miller, 1999). By using a drawing task which assess school-aged children’s cognition (e.g., Nand et al., 2014), we examined whether Mongolian school-aged children and teenagers would show a pattern of drawing styles similar to, or different from analytic/object-oriented/independent North Americans (e.g., European Canadians and European Americans) or holistic/context-oriented/interdependent East Asians (e.g., Japanese). We also examined whether Mongolian children and teenagers would draw any particular objects (e.g., houses, clothing, animals, scenic images) which may represent unique characteristics of Mongolian culture.

### Materials and Methods

#### Participants

A total of 334 school-aged children and teenagers from Grade 1 through Grade 12 at Shine Mongol School in Ulaanbaatar, Mongolia, participated in the current study. The participants consisted of 23 first graders (13 females, eight males, two undeclared), 25 second graders (12 females, 13 males), 29 third graders (14 females, 14 males, one undeclared), 28 fourth graders (18 females, 12 males), 30 fifth graders (15 females, 15 males), 27 sixth graders (13 females, 14 males), 27 seventh graders (11 females, 16 males), 22 eighth graders (11 females, 11 males), 25 ninth graders (14 females, 11 males), 30 tenth graders (13 females, 17 males), 39 eleventh graders (22 females, 17 males), and 29 twelfth graders (21 females, 8 males). An additional 14 students were tested but were not included in our analyses due to the omission of necessary items. The majority of students were born in Mongolia, but 20 students were born abroad (eight in Japan, three in the United States, two in Australia, two in the United Kingdom, one in Korea, one in China, one in Singapore, one in Canada, and one in Germany). All students understood the instructions in Mongolian, and data from these students made up only 5.9% of the total data; therefore, we included their data into our final analyses.

### Materials and Procedure

The materials and procedure were identical to those used in the previous studies (Nand et al., 2014; Senzaki et al., 2014). Students who participated in the study were provided with a pencil and a 392 mm × 271 mm sheet of paper on which to draw a landscape picture. The study was conducted in a classroom setting, where a male teacher, who is the second author, accompanied by the first author, gave instructions in Mongolian. It was emphasized that there was no right or wrong way to draw the picture, and that the children were free to complete their own artwork as they wished. The classroom teachers were sometimes present to observe the session and help with collecting students’ artwork, but they did not administer the session. After instruction and their consent to participate, the students were asked to draw a landscape image including a human, house, tree, and a horizon. They were also told that they could incorporate any other additional objects into their pictures. To standardize students’ understanding of the concept of horizon, the teacher explained what a horizon is, using the following instruction: “When you go outside, you see that the sky comes down and meets the ground, and makes one line. That line is called a horizon.” The total time of the session including instruction and data collection was 30 min.

### Results

#### Types of Drawings with Horizon Lines

Based on Senzaki et al.’s (2014) coding scheme, we measured the top and bottom parts of the horizon drawn by each student. We then computed the average of the two values as the artwork’s location of horizon, and the ratio of the horizon height against the entire frame height. A research assistant and the third author independently measured the location of the horizon. Intercoder agreement in measuring these values were *r* = 0.995. For the random 30% of the data, the intraclass correlation coefficient (ICC) between the two coders was ICC = 0.976, [95% CI = 964; 984], indicating an excellent reliability. Disagreement was resolved through discussion.

**FIGURE 1:**
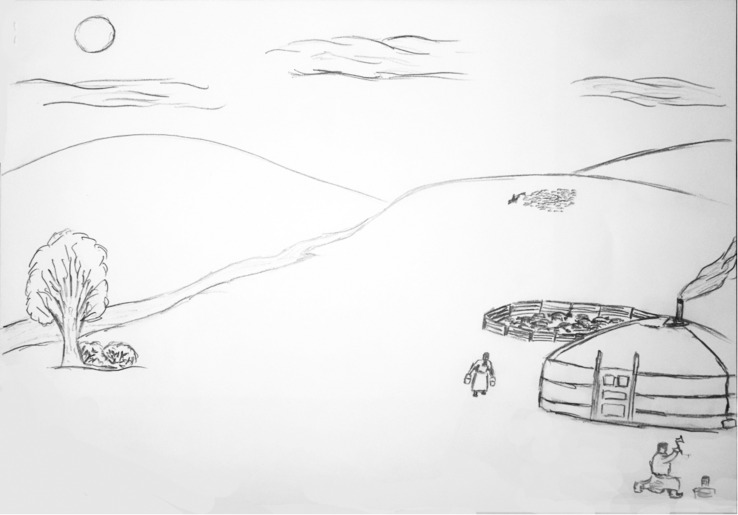
A sample of a Mongolian participant’s landscape drawing.

While the majority of students drew a conventional landscape image, a small number of students produced other patterns of drawings (see Figure 1). For example, in some illustrations, a single horizon was drawn but objects were floating above the horizon (coded as “pictures with floating objects”). In these cases, the height of that single line was measured as the location of the horizon. If children drew an air-gap line (a second line in the sky in addition to the line representing the ground), only the ground line was measured (coded as “pictures with air-gap”). If children used the bottom edge of the paper as a horizon line and placed objects on it, a value of 0 (i.e., a height of 0 cm) was given for the location of the horizon (bottom edge line). Lastly, if children drew only floating objects, we categorized the image as having no horizon line (no horizon) and this data was excluded from the analysis. As seen in Table 1, these patterns are consistent with Canadian and Japanese data (Nand et al., 2014; Senzaki et al., 2014). The universality of this pattern has been addressed in previous papers (Eisner, 1967; Lewis, 1990; Cox and Chapman, 1995; Toku, 2001).

**TABLE 1:** Grade distributions of understanding the concept of horizon.

	Grade distributions													
	*n*	%	1	2	3	4	5	6	7	8	9	10	11	12
Conventional horizon	309	87.5	12	17	23	28	30	27	27	22	25	30	39	29
Floating objects	12	3.4	4	5	2	0	0	0	0	0	0	1	0	0
Air gap	15	4.2	7	5	3	0	0	0	0	0	0	0	0	0
Bottom edge line	3	0.8	2	1	1	0	0	0	0	0	0	0	0	0
No horizon	14	4.0	0	2	0	1	0	1	0	0	1	3	6	0

#### Location of Horizon

First, we carried out the ANOVA’s below by including the gender of the participants as one of individual variables. However, there were no main effects or interaction of gender and other independent variables. Therefore, we collapsed this variable in the analyses reported below.

**FIGURE 2:**
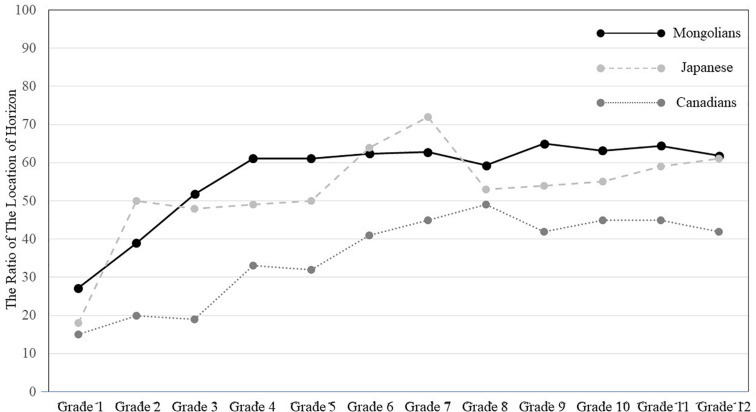
The ratio of horizon height to the entire frame height.

By taking the participants’ grade as an independent variable, a one-way ANOVA was applied to the ratio of the location of the horizon against the entire frame. As shown in Figure 2, there was a significant main effect of grade, *F*(1, 327) = 11.97, *p* < 0.001, η*_*p*_^2^* = 0.290. The results of multiple *t* tests indicated that as children grow older, the location of the horizon in their drawings becomes higher. For example: Grade two’s location of horizon (*M* = 0.40, *SD* = 0.22) was significantly higher than that of Grade one’s (*M* = 0.27, *SD* = 0.22), *t*(322) = 2.82, *p* < 0.01; Grade three’s location of horizon (*M* = 0.54, *SD* = 0.29) was significantly higher than that of Grade two’s, *t*(322) = 3.07, *p* < 0.01. However, Grade four’s location of horizon (*M* = 0.61, *SD* = 0.14) did not significantly differ from that of Grade three’s, *t*(322) (1.54, ns; and the location of horizon of Grade five (*M* = 0.610.61, *SD* = 0.150.15), Grade six (*M* = 0.620.62, *SD* = 0.160.16), Grade seven (*M* = 0.62, *SD* = 0.09), Grade eight (*M* = 0.59, *SD* = 0.08), Grade nine (*M* = 0.65, *SD* = 0.09), Grade ten (*M* = 0.63, *SD* = 0.18), Grade eleven (*M* = 0.64, *SD* = 0.15), and Grade twelve (*M* = 0.63, *SD* = 0.16) did not significantly differ from each other, *t*(322) < 1, ns.

The general patterns of the location of the horizon from Grade one through twelve seem to be identical to that of Canadian and Japanese school-aged children and teenagers reported in Nand et al. (2014) and Senzaki et al. (2014). However, it is important to scrutinize cultural variations in the artworks by comparing the patterns across cultures in detail. We merged the current data with those of previous studies (Nand et al., 2014). A 3 (Culture: Mongolians, Canadians, and Japanese) × 12 (Grade: Grade one through Grade twelve) was applied to the ratio of the location of the horizon against the entire frame in order to examine cultural variations in the artworks. The results indicated that there was a main effect of culture, *F*(2, 1192) = 117.73, *p* < 0.001, η*_*p*_^2^* = 0.169. In general, the location of horizon in Mongolian data (*M* = 0.58, *SD* = 0.19) is higher than that of Japanese (*M* = 0.53, *SD* = 0.27), *t*(1192) = 2.84, *p* < 0.01, and Canadian’s data (*M* = 0.35, *SD* = 0.21), *t*(1192) = 15.12, *p* < 0.001. There was also a main effect of grade, *F*(11, 1193) = 29.85, *p* < 0.001, η*_*p*_^2^* = 0.221. Overall, the location of the horizon in drawings became higher with the participant’s increased grade level: Grade two’s location of horizon (*M* = 0.38, *SD* = 0.300.30) was significantly higher than that of Grade one (*M* = 0.18, *SD* = 0.20), *t*(1192) = 5.89, *p* < 0.001; Grade three’s location of horizon (*M* = 0.37, *SD* = 0.29) did not differ from that of Grade two, *t* < 1, ns; Grade four’s location of horizon (*M* = 0.46, *SD* = 0.23) was significantly different from that of Grade three, *t*(327) = 3.15, *p* < 0.001; Grade five’s location of horizon (*M* = 0.46, *SD* = 0.23) did not differ from that of Grade four, *t* < 1, ns; Grade six’s location of horizon (*M* = 0.55, *SD* = 0.24) was higher than that of Grade five, *t*(1192) = 3.23, *p* < 0.001; and the location of horizon of Grade seven (*M* = 0.58, *SD* = 0.17), Grade eight (*M* = 0.53, *SD* = 0.16), Grade nine (*M* = 0.55, *SD* = 0.19), Grade ten (*M* = 0.55, *SD* = 0.20), Grade eleven (*M* = 0.59, *SD* = 0.16), and Grade twelve (*M* = 0.56, *SD* = 0.19) did not significantly differ from each other, *t*s(1192) < 1.50, ns.

More importantly, there were interactions between culture and grade levels, *F*(22, 1192) = 2.93, *p* < 0.001, η*_*p*_^2^* = 0.053 (see Figure 2). The location of horizons drawn by all grades of Mongolians was higher than that of Canadians: Grade one: *t*(1192) = 2.44, *p* < 0.01; Grade two: *t*(1192) = 4.00, *p* < 0.001; Grade three *t*(1192) = 2.44, *p* < 0.01; Grade four: *t*(1192) = 2.44, *p* < 0.01; Grade five: *t*(1192) = 2.44, *p* < 0.01; Grade six: *t*(1192) = 2.44, *p* < 0.01; Grade seven: *t*(1192) = 2.44, *p* < 0.01; Grade eight: *t*(1192) = 2.44, *p* < 0.01; Grade nine: *t*(1192) = 2.44, *p* < 0.01; Grade ten: *t*(1192) = 2.44, *p* < 0.01; Grade eleven: *t*(1192) = 2.44, *p* < 0.01; and Grade twelve: *t*(1192) = 2.44, *p* < 0.01. As for the comparison between Japanese and Mongolian data, there were a few significant differences in the location of the horizon. First, the location of horizons drawn by Mongolian children and teenagers was higher than that of their Japanese counterparts only in Grade one: *t*(1192) = 2.08, *p* < 0.05; Grade five: *t*(1192) = 2.54, *p* < 0.01; Grade six: *t*(1192) = 2.44, *p* < 0.01; Grade nine: *t*(1192) = 1.99, *p* < 0.05; and Grade ten: *t*(1192) = 2.44, *p* < 0.01. The location of horizons drawn by Japanese was higher than that of their Mongolian counterparts in Grade two, *t*(1192) = 2.33, *p* < 0.02, and there were no statistical significances in the location of horizons in Grades three, four, seven, eight, eleven, and twelve, *t*s(1192) < 1.60, ns. In sum, the cross-cultural comparisons among Canadians, Japanese, and Mongolians allowed us to conclude that the pattern of Mongolian data was quite similar to that of Japanese data, but substantially different from Canadian data.

#### Types of Houses

**FIGURE 3:**
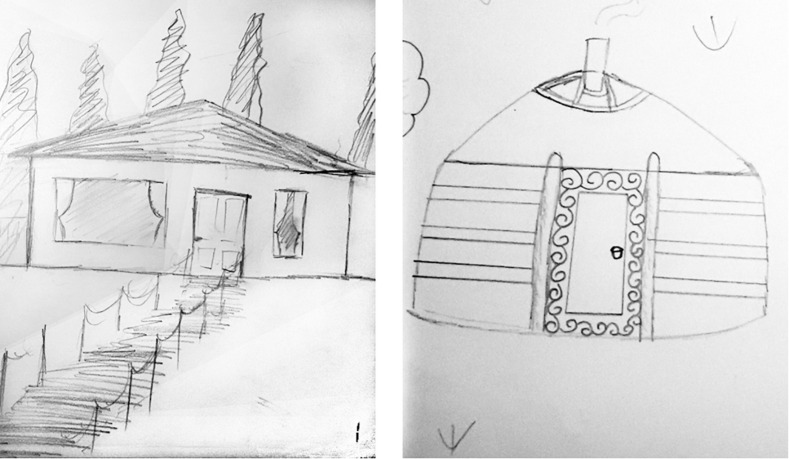
Samples of traditional vs. modern houses drawn by Mongolian participants.

In addition to the cross-cultural comparisons regarding the locations of horizons, we observed several unique drawing styles in the Mongolian data. One aim of the current paper was to emphasize the importance of overcoming the WEIRD issue in psychological research (e.g., Henrich et al., 2010) and extend away from the East vs. West framework (e.g., [Bibr B36][Bibr B28]). In order to do this, we first focused on the types of houses. Historically, Mongolian houses are a round shaped portable tent called *a ger*, made of wood and felt, a textile material that is produced by matting, condensing, and pressing fiber of sheep wool. We examined whether children and teenagers drew modern houses (e.g., Western style houses) or traditional *ger* houses (see Figure 3). Participants were asked to draw at least one house in their landscape as one of the required objects to complete their drawing. In the analysis phase, we assigned a value of one for each type of house depicted in the drawing. For example, if a participant drew two traditional houses and a modern house in his/her artwork, we assigned 1, 1, respectively. If they drew a traditional house but did not draw a modern house, we assigned 1, 0, respectively.

**FIGURE 4:**
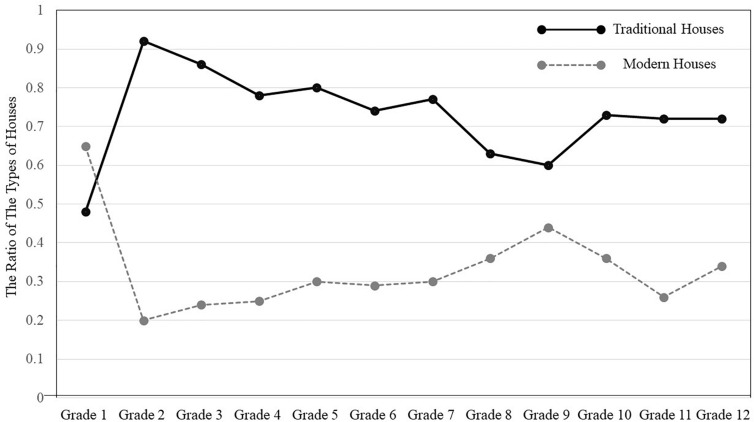
The ratio of traditional vs. modern houses.

Next, we contrasted whether there were any trends in the types of houses, by carrying out a 2 (Types of Houses: traditional vs. modern) × 12 (Grade: one through twelve) ANOVA, treating types of houses as a repeated measure. The results indicated that there was a main effect of types of houses, *F*(1, 322) = 74.21, *p* < 0.001, η_*p*_^2^ = 0.187. Overall, Mongolian children and teenagers were more likely to draw traditional houses (*M* = 0.73, *SD* = 0.44) than modern houses (*M* = 0.33, *SD* = 0.47). There was an interaction between types of houses and grade as well, *F*(1, 322) = 1,89, *p* = 0.04, η*_*p*_^2^* = 0.061. The results of multiple *t* tests indicated that participants drew traditional houses more than modern houses in Grade two, *t*(322) = 5.87, *p* < 0.001; Grade three, *t*(322) = 4.59, *p* < 0.001; Grade four, *t*(322) = 3.57, *p* < 0.001; Grade five, *t*(322) = 3.34, *p* < 0.005; Grade six, *t*(322) = 2.73, *p* < 0.05; Grade seven, *t*(322) = 2.94, *p* < 0.01; Grade ten, *t*(322) = 2.25, *p* < 0.05; Grade eleven, *t*(322) = 3.65, *p* < 0.001; and Grade twelve, *t*(322) = 2.37, *p* < 0.05, but the pattern did not reach statistical significance in Grade eight, *t*(322) = 1.30, ns, and Grade nine, *t* < 1, ns. Finally, the pattern was reversed in Grade one but again did not reach statistical significance, *t* < 1, ns (see Figure 4).

#### Types of Clothes

**FIGURE 5:**
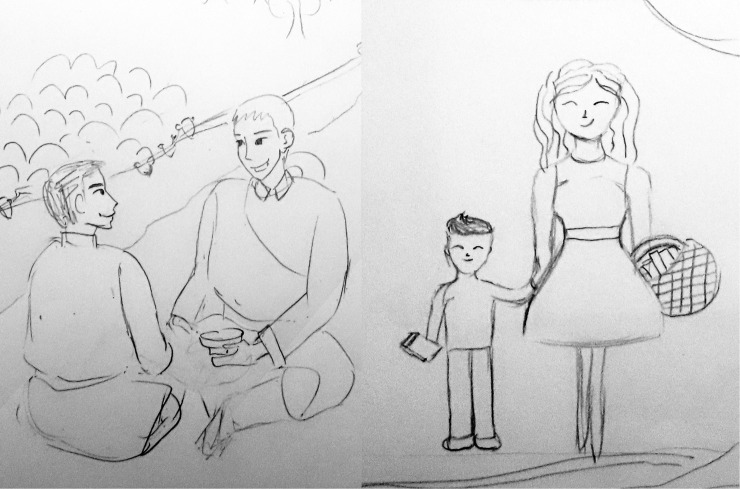
Samples of traditional vs. modern clothes drawn by Mongolian participants.

**FIGURE 6:**
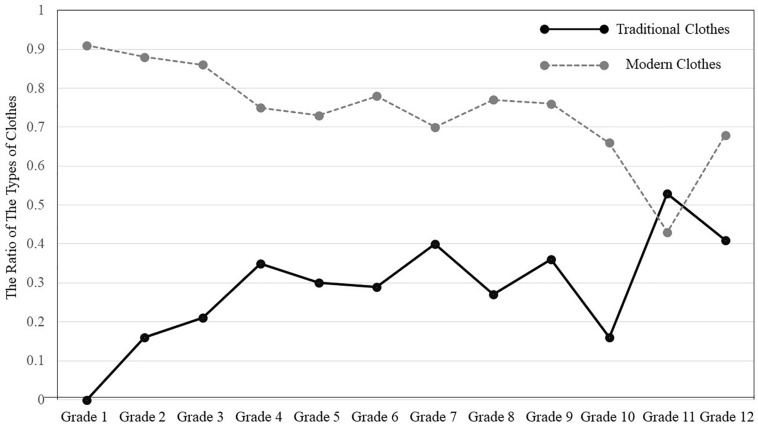
The ratio of traditional vs. modern clothes.

Second, we examined whether children and teenagers drew people with modern clothes (e.g., Western style clothes) or traditional clothes called *deel* (see Figure 5). Participants were asked to draw at least one person in the landscape image. Types of clothes were categorized into two types: modern vs. traditional. Similar to the analyses in the previous section, we assigned a value of one for each type of clothing observed. For example, if they drew a person who wore traditional clothes and two people who wore modern clothes, we assigned 1, 1, respectively. If they drew a person with traditional clothes but did not draw a person with modern clothes, we assigned 1, 0, respectively. Then we contrasted whether there were any trends in the types of clothes, by carrying out a 2 (Types of Clothes: traditional vs. modern) × 12 (Grade: one through twelve) ANOVA, treating types of houses as a repeated measure. The results indicated that there was a main effect of types of clothes, *F*(1, 322) = 110.29, *p* < 0.001, η*_*p*_^2^* = 0.255. Overall, Mongolian children and teenagers were more likely to draw people with modern clothes (*M* = 0.73, *SD* = 0.44) than traditional clothes (*M* = 0.30, *SD* = 0.46). There was an interaction between types of clothes and grade as well, *F*(1, 322) = 3,18, *p* < 0.001, η*_*p*_^2^* = 0.098. The results of multiple *t* tests indicated that participants drew modern clothes more than traditional clothes in Grade one, *t*(22) = 15.20, *p* < 0.001; Grade two, *t*(24) = 5.31, *p* < 0.001; Grade three, *t*(28) = 5.27, *p* < 0.001; Grade four, *t*(27) = 2.50, *p* < 0.02; Grade five, *t*(29) = 2.90, *p* < 0.01; Grade six, *t*(26) = 3.32, *p* < 0.005; Grade eight, *t*(21) = 2.93, *p* < 0.01; Grade nine, *t*(24) = 2.31, *p* < 0.05; and Grade ten, *t*(29) = 3.75, *p* < 0.001; however, this pattern did not reach statistical significance in Grade seven, *t*(26) = 1.77, 0.05 < *p* < 0.10; Grade eleven, *t* < 1, ns; or Grade twelve, *t*(29) = 1.68, *p* > 0.10 (See Figure 6).

#### Types of Animals

**FIGURE 7:**
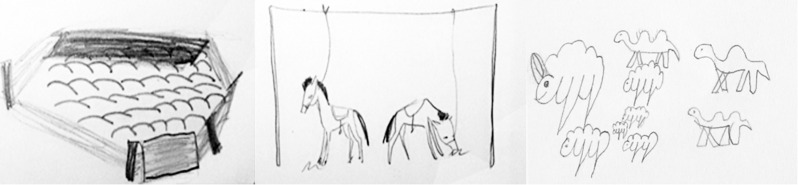
Samples of animals drawn by Mongolian participants.

**FIGURE 8:**
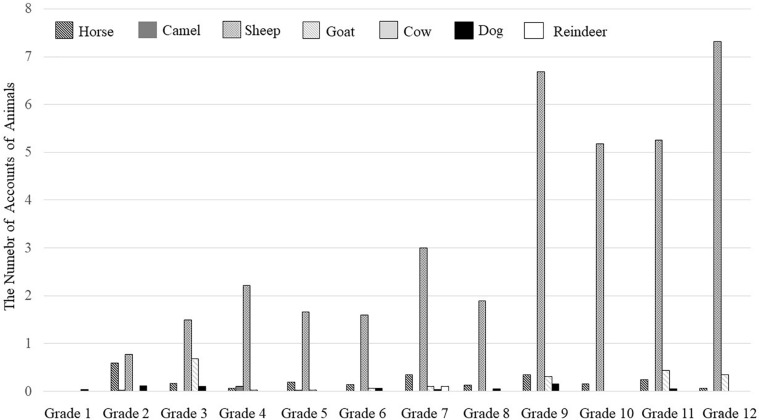
The number of accounts of seven types of animals: Horses, camels, sheep, goats, cows, dogs, and reindeer.

Although it was not required, many Mongolian children and teenagers drew animals including sheep, horses, goats, dogs, cows, camels, and reindeer as additional objects in their artwork (see Figure 7). We assume that the large number of animal images is due to the participants’ familiarity with herding lifestyles. Historically, Mongolians have lived with five animals including horses, sheep, goats, camels, and cows. Additionally, dogs are seen as useful domesticated animals which serve as shepherds, and reindeer have been domesticated in Western and Northern Mongolia, but are not as popular in Central, South, and East Mongolia. We targeted these seven animals since they were most commonly drawn. A 7 (Types of Animals: Sheep, Horse, Goat, Dog, Cow, Camel, and Reindeer) × 12 (Grade one through twelve) ANOVA was carried out, using the number of Types of Animals as a repeated measure (see Figure 8). The results indicated that there was a main effect of types of animals, *F*(1, 322) = 34.34, *p* < 0.001, η_*p*_^2^ = 0.096. Overall, Mongolian children and teenagers draw more sheep (*M* = 3.00, *SD* = 9.81) than horses (*M* = 0.21, *SD* = 0.61), *t*(322) = 5.25, *p* < 0.001, camels (*M* = 0.01, *SD* = 0.18), *t*(322) = 5.56, *p* < 0.001, goats (*M* = 0.05, *SD* = 0.33), *t*(322) = 5.51, *p* < 0.001, cows (*M* = 0.03, *SD* = 0.28), *t*(322) = 5.54, *p* < 0.001, dogs (*M* = 0.05, *SD* = 0.22), *t*(322) = 5.49, *p* < 0.001, and reindeer (*M* = 0.01, *SD* = 0.16), *t*(322) = 5.57. *p* < 0.001. The second most popular animal in their drawings were horses. They drew more horses than camels, *t*(322) = 5.58, *p* < 0.001, goats, *t*(322) = 4.63, *p* < 0.001, cows, *t*(322) = 4.98, *p* < 0.001, dogs, *t*(322) = 4.63, *p* < 0.001, and reindeer, *t*(322) = 5.79, *p* < 0.001. Goats and dogs were more popular than reindeer, *t*(322) = 2.07, *p* < 0.05 and *t*(322) = 2.77, *p* < 0.01, respectively. Other comparisons among animals did not reach statistical significance.

There was an effect of grade, *F*(11, 322) = 2.16, *p* = 0.017, η*_*p*_^2^* = 0.069, but it should be qualified by the interaction between types of animals and grade as well, *F*(11, 322) = 1.99, *p* = 0.029, η*_*p*_^2^* = 0.064. The results of multiple *t*-tests indicated that as children and teenagers’ grades went up, they drew more sheep. Young school-aged children rarely drew sheep, and the values were significantly lower for Grade one (*M* = 0.00, *SD* = 0.00) participant’s when compared to Grade seven (*M* = 3.11, *SD* = 6.19), *t*(322) = 3.01, *p* < 0.001, Grade two (*M* = 0.20, *SD* = 0.81), *t*(322) = 2.88, *p* < 0.001, Grade three (*M* = 0.48, *SD* = 1.50), *t*(322) = 2.70, *p* < 0.001, and Grade four (*M* = 0.46, *SD* = 2.26), *t*(322) = 2.70, *p* < 001, but there were no differences between Grade five (*M* = 1.67, *SD* = 7.50) and seven (*M* = 3.11, *SD* = 6.19), *t*(322) = 1.49, ns, and between Grade six (*M* = 1.67, *SD* = 4.67) and seven, *t*(322) = 1.45, ns. However, there were significant gaps in values between Grade seven and nine (*M* = 6.16, *SD* = 19.97), *t*(322) = 3.02, *p* < 0.001 as well as Grade eight (*M* = 1.09, *SD* = 2.34) and nine, *t*(322) = 4.77, *p* < 0.001. Grade nine, ten (*M* = 5.70, *SD* = 13.03), eleven (*M* = 6.08, *SD* = 9.04), and twelve (*M* = 7.07, *SD* = 17.57) showed the highest values regarding the number of sheep and the value of Grades, however, we did not observe any trends in other animals, *t*s(322) < 1, *p* < 0.001.

#### Correlational Analyses

We further analyzed associations between the location of the horizon and the number of modern/traditional houses, modern/traditional clothes, and animals drawn in the scene. The results (*N* = 334) indicated that there were significant positive correlations between the location of the horizon and the number of traditional houses (*r* = 0.13, *p* = 0.01), traditional clothes (*r* = 0.21, *p* < 0.001) and sheep (*r* = 12, *p* = 0.03), suggesting that traditional Mongolian cultural resources may sustain their holistic/context-oriented/interdependent cognition, rather than analytic/object-oriented/independent cognition.

The number of traditional houses positively correlated with the number of traditional clothes (*r* = 0.21, *p* < 0.001), horses (*r* = 0.27. *p* < 0.001), sheep (*r* = 0.21, *p* < 0.001), and goats (*r* = 0.15, *p* < 0.006). The number of traditional clothes also positively correlated with the number of horses (*r* = 0.17, *p* = 0.002) and goats (*r* = 0.12, *p* = 0.034). In contrast, the number of modern houses negatively correlated with the number of traditional houses (*r* = −0.28, *p* < 0.001), and horses (*r* = 11, *p* = 046), but positively correlated with the number of modern clothes (*r* = 0.26, *p* < 0.001). The number of modern clothes negatively correlated with the number of traditional clothes (*r* = −0.30, *p* < 0.001), but positively correlated with the number of modern houses (*r* = 0.26, *p* < 0.001). These findings suggest that there was clear consistency among the objects drawn by Mongolian school-age children: the more they drew traditional objects, the less they drew modern objects, and vice-versa, suggesting that they still have rich and tangible sources for drawing traditional scenes in modern Mongolia, and that they conceptually distinguish traditional objects from modern objects.

For drawings of animals, the number of horses positively correlated with the number of sheep (*r* = 0.20, *p* < 0.001) and goats (*r* = 0.22, *p* < 0.001). The number of camels positively correlated with the number of goats (*r* = 0.14, *p* < 0.011). Finally, the number of sheep positively correlated with the number of goats (*r* = 0.11, *p* < 0.042). These findings suggest that Mongolian school-age children completed the assigned drawing with rich sources from herding culture, when they drew such countryside scenes.

#### Types of Mountains

**FIGURE 9:**
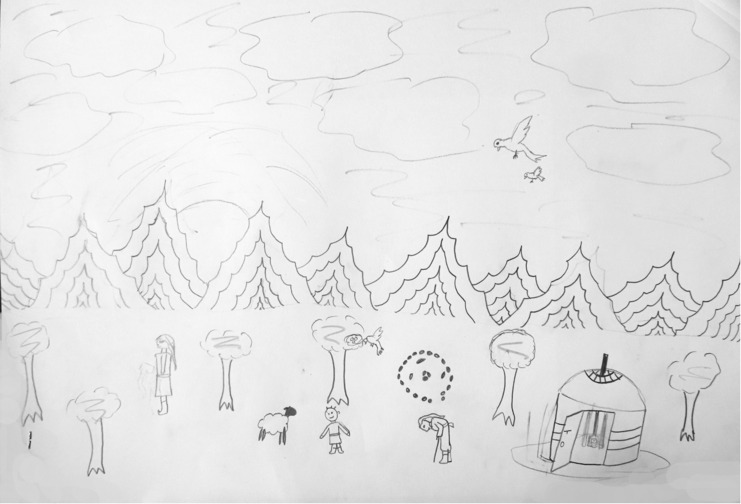
A sample image with a culturally specific mountain drawing.

Finally, there was one more unique characteristic in Mongolian children and teenagers’ drawings worth mentioning (Table 2). A small number of participants across almost all grades drew a multi layered chain of mountains (Figure 9). This style of drawing is observable in conventional artworks and images in the city. However, no Canadian or Japanese participants drew mountain chains in such a way. This implies that Mongolian children and teenagers were exposed to a specific visual representation accessible in their society and internalized the techniques of drawing images in accordance to the culturally shared tradition. Further investigation into the transmission of cultural representations of artwork is required.

**TABLE 2:** Grade distributions of culture-specific mountain drawings.

	Grade distributions													
	*n*	%	1	2	3	4	5	6	7	8	9	10	11	12
Mountain drawing	21	6.2	3	2	2	4	1	2	0	1	1	2	2	1
Total	334	100.0	23	25	29	28	30	27	27	22	25	30	39	29

### Discussion

The current case study examined Mongolian children and teenagers’ landscape artwork. The results of local comparisons of Mongolian data, and cross-cultural comparisons between Mongolians, Canadians, and Japanese provided us with a variety of new knowledge. First of all, similar to their Canadian and Japanese counterparts, young Mongolian children (e.g., Grade one) captured three-dimensional scenes by placing the horizon in a lower section of the frame. But the location of the horizon gradually moved up as their grade level increased. Finally, a culturally dominant way of landscape drawing styles stabilized around Grades four to five, and became constant in their teenage years. Overall, the results of the Mongolian data were more similar to that of Japanese than to Canadians, suggesting that, while being exposed to their pastoral social orientation (which has been discussed as the antecedent of analytic/object-oriented/independent cognition), Mongolian children and teenagers were more likely to be influenced by their holistic/context-oriented/interdependent cognition when they drew landscape images (e.g., Masuda, 2017; Masuda et al., 2019).

Beyond the discussions of universality and cultural specificity of landscape drawing, the current study also provided us with rich information on Mongolian children and teenagers’ life experiences. For example, while the school is situated in the center of Ulaanbaatar, the capital city of Mongolia, where Western style houses and buildings are dominant, many children in fact have had the opportunity to visit the countryside and view traditional *ger* houses. We assume that such experiences provided an image of what a unique Mongolian landscape looks like. Therefore, when they were asked to complete the landscape drawing, Mongolian children and teenagers seem to accurately represent traditional *ger* houses. In contrast, though traditional Mongolian clothes are popular for attending special events such as ritual ceremonies and festivals, modern clothes are dominant both in cities and the countryside. We assume that when Mongolian children and teenagers were asked to draw a landscape drawing, they reflected on images of familiar clothing for their drawings. However, it is interesting that such a tendency weakened as they became teenagers, potentially implying that adolescents think the traditional *deel* clothes play an important role in identifying themselves as mature Mongolians. Future studies should further investigate the relationship between cultural traditions and identity.

In terms of the types of animals, the data showed that sheep and horses were two popular animals in the landscape drawings. The number of sheep drawn increased gradually as children attended higher grades. These results may reflect the fact that older children and teenagers were more aware of the importance of the pastoral economy in Mongolia as well as the fact that horses are an important symbol that represent this society, or it may just reflect the fact that older children are generally more skilled at drawing. The fact that the number of sheep are larger than any of the other animals may reflect that sheep are dominantly popular around the village: compared to horses and cows which search for grass far from the village, goats and sheep roam the grassland closer to the village, which allow them to be viewed more frequently. Furthermore, the ratio of goats to sheep in this area is traditionally 1:10. As a result, herds of sheep are frequently viewed by Mongolians, which can explain their patterns of drawings. The current results may accurately represent reality for Mongolians.

The findings based on correlational analyses further give credence to the connection between their holistic/context-oriented/interdependent drawing style and their motivation to draw traditional resources such as traditional *ger* and *deel*. The number of sheep also supports this connection. One may question why the pattern of cognition shared by this traditionally herding-oriented culture appears to contradict with that of other herding societies (e.g., Uskul et al., 2008). Fijn’s (2011) observation in her book “Living with Herds: Human-Animal Co-existence in Mongolia” may provide some insights. In her book, she observed that Mongolians’ pictorial depiction of their homeland captures the whole range of valley and mountain in their drawing as if they perceive the connectedness of all elements (p. 58), suggesting that it is natural for them to sustain holistic/context-oriented/interdependent tendencies. Future research should investigate possibilities of more nuanced differences in cognition among these traditional herding cultures.

#### Limitations

While we maintain that the cultural variations in drawing styles could be explained by the framework of holistic vs. analytic cognition, there remain other possibilities which affect Mongolian school age children’s drawing patterns, such as their perceived social expectations regarding their performance, and their intentions on what they want to communicate to others. For instance, their sense of social desirability to express their cultural identity may direct them to draw more traditional objects rather than modern objects in the scenes. By adding other measures, future studies need to mitigate such possible biases.

It is also noteworthy that several years have passed between the collection of the current Mongolian data and previous studies targeting Canadians and Japanese. While we maintain that patterns of cognition do not change quickly, and the data would be replicable if we were able to collect Canadian and Japanese data again, it is prudent to address this limitation. Future research should elucidate this point.

Finally, the current study assumed that there is a sensitive period for school-age children to develop culturally specific patterns of responses, and the patterns we observed are similar to those of young adults in previous studies (Nand et al., 2014; Senzaki et al., 2014). Therefore, it was crucial for the current study to select school-age children as subjects and match their age range with the previous studies. We examined whether there are any trends regarding how they draw images, and whether the patterns are similar to holistic Japanese school-age children’s data or analytic Canadian school-age children’s data. While we argue that this preliminary investigation successfully controlled for age factors, and replicated the effectiveness of the methodology, it is also prudent to further examine whether the patterns are actually sustained among young adults, middle-aged, and elder people in Mongolia. By using this useful drawing task, we also plan to examine potential within-culture variations between those who live in cities and those who live in the countryside, such as mountainous and desert areas.

## General Discussion

The current paper addressed the importance for cultural psychologists to target research fields outside of G7 and G20 countries, while going beyond the constraints of the WEIRD cultures and the East/West dichotomy. While reviewing classic cross-cultural studies in the 1960s, we discussed the similarities between them and contemporary cultural psychology. At the same time, we addressed the field’s need to evolve by focusing more on the internalization and socialization processes of a given culture’s meaning system (Bruner, 1990; Shweder, 1991; Miller, 1999) in order to elucidate the impact of culture on human cognition. The motivation to search for universal phenomena is inherent in scientific investigation, but we may too easily accept equivalency in cognition across cultures without a broad enough sample set outside of North America. Given recent discourse on the pros and cons of globalization, now is a good time to expand our research in order to scientifically define culture’s role on cognition—and the actions we take that have global scope. We hope that our paper will motivate scholars to break through existing constraints in our field.

Here, we include a case study from Mongolia, and compare the data with previous cross-cultural findings from Japan and Canada (Nand et al., 2014; Senzaki et al., 2014). The case study had several implications. First, it demonstrated a possible way of extending established research to a new cultural site, Mongolia. Second, the addition of new cultural data suggested universal patterns of human development with regards to the concept of horizon. For example, there appears to be a general trend in which the pictorial representation of the location of the horizon gradually rises across 12 years of schooling. Third, by incorporating the current data into the existing dataset (Nand et al., 2014), we identified cultural similarities and differences between Mongolians, Japanese, and Canadians. These findings allow us to speculate the causes of cultural variation in drawing styles such as the level of interdependence, holistic thinking style, and specific aspects of herding traditions shared in Mongolia. Future studies must investigate the causes of cultural variations in order to understand the mutual relationship between culture and the human mind.

### How to Conduct Research in a New Culture

In addition to presenting our case study, we would like to share our experiences in conducting cultural research. Here, we will discuss some potential challenges along with possible solutions, in hopes that they will inform new cultural researchers. Some of these points have been already discussed in anthropology and cultural studies, yet we maintain it is worth sharing them with contemporary cultural psychologists who are interested in broadening their scope of research.

#### Respect the Target Culture’s Rules and Customs

Each culture has its own rules and customs for communicating and creating interpersonal relationships with people. Although researchers may be more comfortable following their own rules and customs, it is important for them to accommodate the guidelines of the target culture. This message is especially important for researchers studying basic psychological processes who have collected data from participants only in their cultural community, because, in most cases, they would be unaware of substantial cultural variations in human behaviors in general. We strongly urge these researchers to expose themselves to new cultural experiences and deeply learn about the target culture and its language. Such observational and anthropological knowledge will be the foundation of their research ideas.

#### Find a Way to Thank Local Collaborators

Finding collaborators in a new culture is indeed time-consuming and laborious. Once you create a relationship with them, it is necessary for researchers to find a way to thank them, both in the short-term and the long-term. Benefits for the collaborators could include direct monetary support or official recruitment of paid assistants. However, there are also many indirect benefits. For example, if you collect data at a high school, the local collaborators may request you to give a series of lectures, to establish exchange programs, or even collect data from your own culture. Unidirectional relationships can be destructive, so it is important to search for long-term mutual benefits after the data collection is complete, so that a positive relationship can be formed between cultures.

#### Spend Enough Time Discussing Research Ethics

While collecting data under the institution to which you belong, the ethics board requires you to follow the board’s cultural ethics and rules. In some cultures, however, there might not be such a human ethics board, or if there is, the structure and procedure of conducting research might be quite different from yours. In this case, you are required to discuss with both human ethics boards and representatives of the target culture to find a comparable and optimal set of rules to follow. This may include how to get consent and agreement, maintaining participants’ voluntary participation rules and their right to select alternative tasks, withdrawal from the session, confidentiality of participants’ identities, a data withdrawal policy, and permission for researchers to publicly report their findings. The meticulous solution and optimization will be the foundation of your publication in international journals which meet the standard of international academia.

#### Using Easy-to-Administer Research Materials

Beginning with an easy task is a suitable way to collect data from a new target culture for many reasons. First, procedural ease helps the collaborators administer the session, and reduces potential mistakes. Second, the first experience is key for the collaborators to estimate the cost of the research and future collaborations. Third, carrying specific experimental devices and materials are costly for both researchers and collaborators, and may require filling out documents and paying extra fees when going through customs. In general, paper-and-pencil materials are tangible and make it easy for participants to follow the instructions. A successful and positive first experience is essential for developing a long-term relationship between researchers and local collaborators.

#### Sharing the Findings With Local Collaborators

It is the researchers’ responsibility to discuss and get approval from the local collaborators when their paper is accepted by a professional journal. Once the paper is published, it can impact the collaborators’ personal lives, their institution, and even their culture—especially when the paper addresses a core component of their cultural values to which they are strongly associated. Providing collaborators with enough time to consider the costs and benefits of publishing the paper allows both researchers and collaborators to develop confidence and responsibility regarding their research program. If the population size of a given society is small, the impact of a single publication may strongly influence the target society. Continuous observation of researchers’ output may be required, and researchers need to answer the local people’s queries and concerns responsibly.

## Conclusion

This paper was intended to provide readers with advice to help further advance the field of cultural psychology. We believe that acknowledging the long history of research on cultural variations in basic perceptual processes since the 1960s and reviving these lines of research will enrich the discourse of cultural psychology. Through the current study, we shared our experience of starting novel research with collaborators from a school in Ulaanbaatar, Mongolia, and reported the findings. Our future research plans include examining potential variabilities between people living in the countryside and the city, as well as the effect of geographical and vocational variation on Mongolian mentalities. We hope this paper will motivate cultural researchers to go abroad and establish new research sites for advancing cultural psychology.

## Data Availability Statement

The original contributions presented in the study are included in the article/supplementary files, further inquiries can be directed to the corresponding author.

## Ethics Statement

The studies involving human participants were reviewed and approved by Research Ethics Board, University of Alberta; Shine Mongol School. Written informed consent from the participants’ legal guardian/next of kin was not required to participate in this study in accordance with the national legislation and the institutional requirements.

## Author Contributions

TM analyzed the data and wrote a main part of the manuscript. BB administered whole sessions and wrote a part of the manuscript. SS analyzed the data and wrote a part of the manuscript. All authors contributed to the article and approved the submitted version.

## Conflict of Interest

The authors declare that the research was conducted in the absence of any commercial or financial relationships that could be construed as a potential conflict of interest.

## Footnotes

1 The studies involving human participants were reviewed and approved by the Research Ethics Office, University of Alberta. Written informed consent from the participants’ legal guardian/next of kin was not required to participate in this study in accordance with national legislation and institutional requirements.
